# Long noncoding RNA HOXA-AS2 functions as an oncogene by binding to EZH2 and suppressing LATS2 in acute myeloid leukemia (AML)

**DOI:** 10.1038/s41419-020-03193-3

**Published:** 2020-12-02

**Authors:** Yubin Feng, Shuang Hu, Lanlan Li, Xiaoqing Peng, Feihu Chen

**Affiliations:** 1 Inflammation and Immune Mediated Diseases Laboratory of Anhui Province, School of Pharmacy, Anhui Medical University, Hefei, Anhui China; 2 The Key Laboratory of Anti‐inflammatory and Immune Medicines, Ministry of Education, Hefei, Anhui China

**Keywords:** Bone cancer, Bone cancer

## Abstract

Acute myeloid leukemia (AML) is the most common hematological malignancy in the world. Long noncoding RNAs (lncRNAs) play an important role in the development of physiology and pathology. Many reports have shown that lncRNA HOXA cluster antisense RNA 2 (HOXA-AS2) is a carcinogen and plays an important role in many tumors, but little is known about its role in AML. The aim of this study was to explore the potential mechanism and role of HOXA-AS2 in AML. HOXA-AS2 was upregulated in AML cell lines and tissues, and the overexpression of HOXA-AS2 is negatively correlated with the survival of patients. Silencing HOXA-AS2 can inhibit the proliferation and induce differentiation of AML cells in vitro and in vivo. Overexpressing HOXA-AS2 showed the opposite result. Moreover, more in-depth mechanism studies showed that carcinogenicity of HOXA-AS2 exerted mainly through binding with the epigenetic inhibitor Enhancer of zeste homolog 2 (EZH2) and then inhibiting the expression of Large Tumor Suppressor 2 (LATS2). Taken together, our findings highlight the important role of HOXA-AS2 in AML, suggesting that HOXA-AS2 may be an effective therapeutic target for patients with AML.

## Introduction

The two main features of AML are uncontrolled malignant proliferation and blocked differentiation, accounting for 30% of leukemia related deaths^1,2^. With the development of science and technology, the treatment of leukemia has been made great progress, but the cure of the disease is still unsolved, and the 5-year survival rate is only 25%^3–7^. Although AML patients after treatment, the condition has been greatly improved, but the prognosis of many patients is still not ideal. Moreover, the risk of relapse and drug resistance will still occur during chemotherapy, which may become the dilemma of AML treatment^8,9^.

According to the results of human genome research, only a small part of human genes can encode a protein, while the remaining large part of human genome genes (~97%) are tightly transcribed into noncoding RNA^10–12^. As a new noncoding RNA molecule, the abnormal expression of lncRNA in tumor tissue has attracted extensive attention of researchers^13,14^. Many studies have shown that lncRNA is likely to be a carcinogen gene^15,16^ or a tumor suppressor gene^16^ in the occurrence and development of tumors, and has increasingly become a biomarker of tumor diagnosis, treatment, or prognosis, including AML^17,18^. Therefore, it is very important to identify more new cancer-related lncRNAs and explore their biological functions and molecular mechanisms in order to explore new therapeutic methods of AML.

As a 1048-bp lncRNA, HOXA-AS2 has been shown to enhance gastric cancer proliferation through epigenetically silencing the expression of p21, plk3, and DDIT3^19,20^. However, little is known about the role of HOXA-AS2 in the pathogenesis and development of AML. Our results showed that HOXA-AS2 was upregulated in AML cells. TCGA database also showed that HOXA-AS2 expression was also upregulated in AML tissues and was associated with poor prognosis in AML patients. Moreover, silencing of HOXA-AS2 not only inhibited the proliferation and induced differentiation of AML cells but also reduced the tumorigenicity of nude mice. LATS2, as a member of the LATS family located at 13q11-q12^21,22^, mainly regulates cell cycle progression to inhibit tumor occurrence and development^23^. As a member of the Hippo signaling pathway, the loss of LATS2 may destroy the Hippo signaling pathway and eventually lose its tumor-inhibiting function^24^. There have been many reports in the literature that lncRNA can bind to EZH2 to regulate the expression of LATS2^25,26^. We further proved that HOXA-AS2 acts as a modular scaffold for histone-modified complexes by binding with EZH2 to silence LATS2 expression. In conclusion, our results suggest that the HOXA-AS2-EZH2-LATS2 axis may provide a new strategy for the diagnosis and treatment of AML.

## Materials and methods

### Cell lines and culture

NB4 and THP-1 cell lines were provided by Shanghai Gecko gene in 1640 medium containing 10% fetal bovine serum (FBS) (Gibco, United States) containing penicillin (100 U/ml) and streptomycin (100 g/ml) (Gibco, Grand Island, NY, USA). All cells were cultured in a humidified atmosphere, at 37 °C with 5% CO_2_. We changed the cell culture medium every 24 h, and also sub-cultured the cells for 48 h.

### RNA extraction and qRT-PCR assays

Trizol (Invitrogen, USA) was used to extract the total RNA from tissues and cell lines, and then reverse transcripted using Primer-Script One Step RT-PCR kit (TaKaRa, China). Finally, the SYBR premix dimming eraser Kit (TaKaRa, China) was used for real-time RT-PCR detection. GAPDH was used as a standardized control. All measurements were repeated three times. The primer sequences are shown in Table 1.

**Table 1:** Sequences of primers for qRT-PCR and siRNA sequence.

Name	Sequences (5′ to 3′)
*Primers for qRT-PCR*	
HOXA-AS2 (forward)	CCCGTAGGAAGAACCGATGA
HOXA-AS2 (reverse)	TTTAGGCCTTCGCAGACAGC
GAPDH (forward)	AGAAGGCTGGGGCTCATTTG
GAPDH (reverse)	AGGGGCCATCCACAGTCTTC
LATS2 (forward)	ACCCCAAAGTTCGGACCTTAT
LATS2 (reverse)	CATTTGCCGGTTCACTTCTGC
EZH2 (forward)	TGCACATCCTGACTTCTGTG
EZH2 (reverse)	AAGGGCATTCACCAACTCC
*Interference sequences (siRNA)*	
si-HOXA-AS2 #1	GAGUUCAGCUCAAGUUGAACAUACA
si-HOXA-AS2 #2	AAACCUUGUAGAUAGCUUGAGCUGG
siNC	UUCUCCGAACGUGUCACGUTT
siEZH2 #1	GAGGUUCAGACGAGCUGAUUU
siEZH2 #2	AAGACTCTGAATGCAGTTGCT

### Gene knockdown

Small interfering RNA (siRNA) and short-hairpin RNA (shRNA) were used for the knockdown of genes. Lipofectamine 2000 (Invitrogen) was used for transient transfection, and the correlation was performed 48 h after transfection. We purchased HOXA-AS2 siRNA (si-HOXA-AS2) and negative control siRNA (siNC) from General Biology (Chuzhou, China). The primer interference sequences are shown in Table 1.

HOXA-AS2 short-hairpin RNA (LV-sh-HOXA-AS2) and respective negative control (LV-sh-NC) were obtained from General Biology (Chuzhou, China). First, NB4 and THP-1 cells were seeded in 24-well plates before being transfected. Then, 30 ml shRNA was added to each well, allowed to stand at room temperature for 15 min, and placed in a cell culture incubator; and the medium was changed after 24 h. The primer interference sequences are shown in Table 1.

### Gene overexpression

pcDNA-LATS2 vector and pcDNA3.1 vector, as well as pCMV6-XL5-HOXA-AS2, were obtained from General Biology (Chuzhou, China). Amplification efficiencies were determined by qRT-PCR.

### Cell counting kit-8 (CCK8) assays

CCK8 kit was used for detection (Beyotime Institute of Biotechnology, China). The transformed cells were inoculated into a 96-well plate (1 × 10^3^ cells/well), and then detected at 450-nm absorbance every 24 h for 96 h. Five multiple holes were taken for testing each time, and all the assays were conducted in triplicate.

### Cell cycle analysis

The transfected cells were collected and fixed with 70% precooled ethanol. Then, according to the instructions of Shanghai Beibo, biological cell cycle detection kit was used for subsequent operations. Finally, the cell cycle was detected by CytoFLEX (Becton Dickinson, USA) and analyzed by ModFit software.

### Differentiation marker analysis

The detection methods of differentiation indicators (CD11b and CD14) has been presented in our previously published articles^27^.

### Western blotting

We first used 8% SDS-PAGE to isolate the protein lysate, and then transferred the protein to 0.22-µm PDVF membrane. PVDF membrane was incubated with the corresponding primary antibody at 4 °C overnight and then incubated with horseradish peroxidase-labeled secondary antibody at room temperature. All the experiments were repeated three times. β-actin (Cat No. bsm-33036M, Bioss, Beijing, China), rabbit anti-human LATS2 (Cat No. 5888, CST, Boston, USA), rabbit anti-human CDK4 (Cat No. ab108357, Abcam, Danvers, MA, USA), rabbit anti-human Cyclin A2 (Cat No. ab181591, Abcam, Danvers, MA, USA), rabbit anti-human Cyclin D3 (Cat No. ab183338, Abcam, Danvers, MA, USA), rabbit anti-human P-Rb (Cat No. ab184796, Abcam, Danvers, MA, USA), rabbit anti-human CD11b (Cat No. ab133357, Abcam, Danvers, MA, USA), rabbit anti-human CD14 (Cat No. ab133335, Abcam, Danvers, MA, USA), and rabbit anti-human EZH2 (Cat No. ab186006, Abcam, Danvers, MA, USA).

### RNA immunoprecipitation (RIP)

The treated cells were collected, and their nuclear proteins were extracted and then resuspended in RIP buffer. Then divide the resuspended RIP buffer into the input group, IgG group, and EZH2 group. Then collect the supernatant after centrifugation, add IgG (Abcam) or human anti-EZH2 antibody (Abcam), and then incubate at 4 °C for 2 h. Subsequently, protein A beads were added and incubated at 4 °C for 1 h. After centrifugation, the cells were washed three times with RIP buffer and then once with PBS, and the beads were resuspended in Trizol. Finally, quantitative detection was performed by qRT-PCR.

### Luciferase reporter assay

Human LATS2 luciferase reporter gene plasmid was prepared and synthesized by Bioujing bio. The luciferase activities of firefly and marine kidney were measured (Promega, Shanghai, China) consecutively 40–44 h after transfection. Finally, the luciferase activity of the firefly was quantified according to the luciferase standardization. The promoter sequence of LATS2 is as follows:

GCGGGGTCACGTGACGCCCGTGGAATGCCAACAATGTAGCGAATGTCCCACTTGGGTCTG.

### RNA pull-down assay

Biotin-labeled RNA pull-down was performed as described previously^28^. In brief, 1 × 10^7^ AML cells were harvested, lysed, and sonicated. Then, nuclear proteins were extracted using a nuclear plasma-isolation extraction kit and incubated with biotin-labeled HOXA-AS2 truncated probes and streptavidin agarose beads (Invitrogen) at 4 °C overnight. After washing with the wash buffer, the protein was recovered and detected by western blot.

### Chromatin immunoprecipitation (ChIP)

ChIP analysis was carried out according to the manufacturer of the EZ-ChIP kit (Upstate Biotechnology, Lake Placid, NY, USA). The final results were determined by RT-qPCR. The primer sequences are shown in Table 1.

### Fluorescence in situ hybridization (FISH)

Fluorescence in situ hybridization (FISH) was used to detect the location of HOXA-AS2 in the cells. The experimental method was similar to that reported before^27^. The cells were first fixed with 4% paraformaldehyde, then apply the cells on the anti-dropping glass slides to make droplets, then fix and permeabilize the cells, then incubate with FISH probes, stained with DAPI, and finally mount the slides. Fluorescence imaging was analyzed by laser scanning confocal microscope.

### Tumor xenografts

All animal care and experiments were carried out in accordance with the guidance of the National Institutes of Health and approved by the Ethics Committee of Anhui Medical University. We selected 4-week-old NCG mice with severe immunodeficiency for tumor xenotransplantation experiments to study the effect of HOXA-AS2 on tumor proliferation and differentiation. In tumor growth assay in vivo, NB4 cells stably transfected with HOXA-AS2 short-hairpin RNA (LV-sh-HOXA-AS2) and respective negative control (LV-sh-NC) were subcutaneously injected into the upper back of the nude mice (1 × 10^7^, 200 μl). Eight weeks after inoculation, the cells were killed. Finally, mice were sacrificed, and subcutaneous tumor tissues were detected for tumor weight, WB, and IHC staining.

### Immunohistochemistry

Tumor tissue was fixed in 4% paraformaldehyde and embedded in paraffin. After sectioning, it was incubated with CD11b antibody (Bioss, China), CD14 antibody (Bioss, China), Ki67 antibody (Bioss, China), and LATS2 antibody (CST, USA) overnight at 4 °C. Then they were incubated with the second antibody and stained with diaminobenzidine. Photographs were taken with a light microscope.

### Nitroblue tetrazolium (NBT) assay

The transfected cells were inoculated in a six-well plate and were collected. A 10 μl aliquot of NBT solution, composed of 10 mg/ml NBT (Sigma-Aldrich) and 2 μg/ml PMA (Sigma-Aldrich), was added to each well, and then cells were incubated for 30 min at 37 °C. Then, light microscopy was used for analysis. The positive cells ratio was analyzed by light microscopy.

### Statistical analysis

Data were expressed as mean ± standard deviation. One-way analysis of variance (ANOVA) and Duncan test were used for comparison between multiple groups. If the *P* value was <0.05, the difference is considered statistically significant. The experimental results were represented at least three independent experiments.

## Results

### HOXA-AS2 levels were increased in AML

**Fig. 1:**
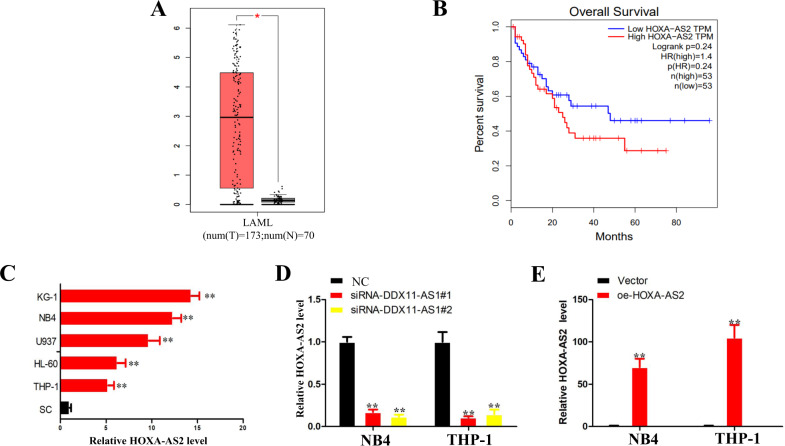
Relative expression of HOXA-AS2 in AML tissues, cells, and its clinical significance. **a** Box plots for HOXA-AS2 gene expression in AML and normal tissues from GEPIA. **b** Kaplan–Meier survival plot of overall survival of AML patients in GEPIA, categorized according to HOXA-AS2 gene expression (high vs. low, based on mean expression). **c** Relative expression levels in leukemia cell lines and SC cells. **d** Relative expression of HOXA-AS2 in NB4 and THP-1 cells transfected with siRNAs. **e** Relative expression of HOXA-AS2 in NB4 and THP-1 cells transfected with HOXA-AS2 plasmid. *P < 0.05, **P < 0.01 versus control groups.

In order to explore the role of HOXA-AS2 in AML, we first used GEPIA database to analyze the expression of HOXA-AS2 in AML patients. The results showed that the expression of HOXA-AS2 in AML tissues (left bar) was significantly higher than that in normal tissues (right bar) (Fig. 1a) (http://gepia.cancer-pku.cn/detail.php). Furthermore, Kaplan–Meier survival analysis showed that the overall survival rate of AML patients with high expression of HOXA-AS2 (*n* = 53) was significantly lower than the patients with low expression of HOXA-AS2 (*n* = 53) (Fig. 1b). qRT-PCR results showed that HOXA-AS2 was highly expressed in many leukemia cell lines (KG-1, NB4, U937, HL-60, and THP-1 cells) compared to the normal human monocytes (Fig. 1c). Based on the above results, we conducted a series of in vitro and in vitro studies to determine the function of HOXA-AS2 in AML.

### HOXA-AS2 regulates AML cell proliferation

**Fig. 2:**
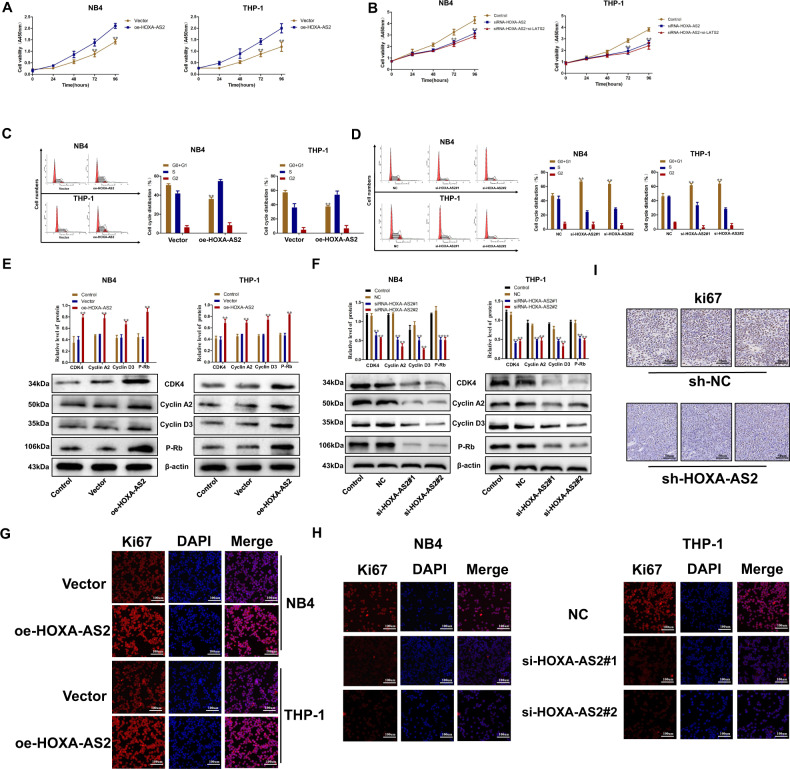
Effect of HOXA-AS2 on AML cell growth. **a**, **b** The proliferation ability of NB4 and THP-1 cells transfected with HOXA-AS2 plasmid or transfected with si-HOXA-AS2#1, si-HOXA-AS2#2 was determined by CCK8 assays. **c**, **d** The cell cycle of transfected NB4 and THP-1 cells. **e**, **f** The levels of cyclin D3, cyclin A2, P-rb, and CDK4 protein in NB4 and THP-1 cells transfected with HOXA-AS2 plasmid or transfected with si-HOXA-AS2#1, si-HOXA-AS2#2 were detected by western blotting assays. **g**, **h** The level of ki67 protein in NB4 and THP-1 cells transfected with HOXA-AS2 plasmid or transfected with si-HOXA-AS2#1, si-HOXA-AS2#2 was detected by immunofluorescence. **i** The ki67 expression of tumors from sh-HOXA-AS2 and sh-NC groups was determined by immunohistochemical staining. *P < 0.05, **P < 0.01 versus control groups.

In order to further explore the biological function of HOXA-AS2 in AML cells, we choose the NB4 and THP-1 cell lines for further studies. The efficiency of silencing and overexpression was verified by qRT-PCR (Fig. 1d, e). si-HOXA-AS2 1# and 2# demonstrated a better silencing capacity. HOXA-AS2 expression was increased in AML cell lines compared with the negative control following transfection with pCMV6-XL5-HOXA-AS2. Thus, we selected si-HOXA-AS2 1# and 2# and pCMV6-XL5-HOXA-AS2 in all following experiments. CCK8 results showed that overexpression of HOXA-AS2 can improve the survival rate of AML cells (Fig. 2a), while silencing HOXA-AS2 has the opposite effect (Fig. 2b). Flow cytometry and western blot were then used to demonstrate the above results. The results of flow cytometry showed that overexpression of HOXA-AS2 expression could inhibit G0/G1 cycle arrest of AML cells, thus promote the proliferation of AML cells (Fig. 2c). On the contrary, inhibition of HOXA-AS2 had the opposite effect in AML cells (Fig. 2d). Overexpression of HOXA-AS2 in AML cells can increase the expression of G0/G1 marker protein (cyclin D3, cyclin A2, P-Rb, and CDK4) (Fig. 2e). In contrast, our results also showed that silencing HOXA-AS2 could induce a decrease of G0/G1 marker protein (Fig. 2f). In order to further confirm the effect of HOXA-AS2 on the proliferation of AML cells, we used immunofluorescence to analyze the expression of Ki67 in the transfected AML cells (Fig. 2g, h). Immunohistochemistry staining showed that silencing HOXA-AS2 inhibited Ki67 expression compared with that in control tumors (Fig. 2i). These results demonstrated that HOXA-AS2 expression levels were associated with AML proliferation.

### HOXA-AS2 regulates AML cell differentiation

**Fig. 3:**
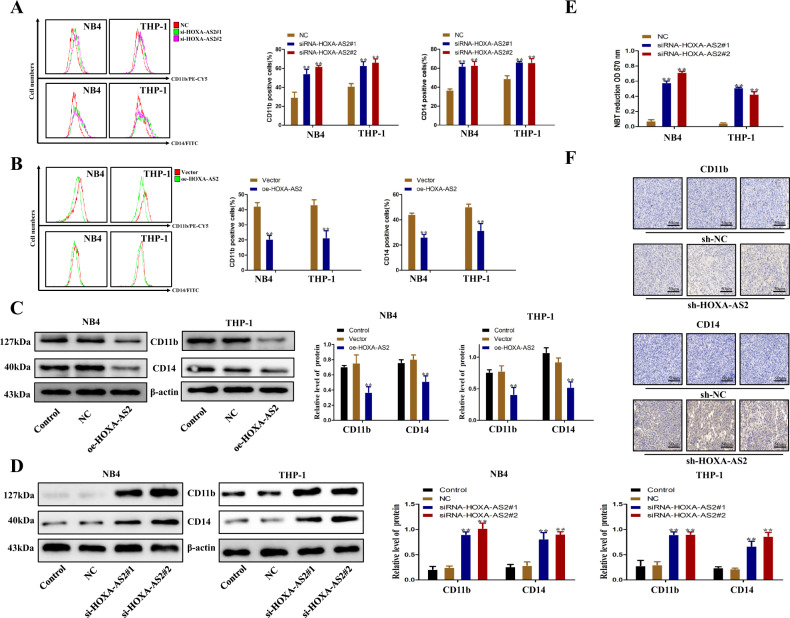
Effect of HOXA-AS2 on AML cells differentiation. **a**, **b** The differentiation ability of NB4 and THP-1 cells transfected with HOXA-AS2 plasmid or transfected with si-HOXA-AS2#1, si-HOXA-AS2#2 was determined by flow-cytometry analysis. **c**, **d** The levels of CD11b and CD14 protein in NB4 and THP-1 cells transfected with HOXA-AS2 plasmid or with si-HOXA-AS2#1 and si-HOXA-AS2#2 were detected by western blotting assays. **e** The differentiation ability of NB4 and THP-1 cells transfected with si-HOXA-AS2#1, si-HOXA-AS2#2 was assessed by evaluating NBT reduction ability. **f** The CD11b and CD14 expression of tumors from sh-HOXA-AS2 and sh-NC groups were determined by immunohistochemical staining. *P < 0.05, **P < 0.01 versus control groups.

Our results have demonstrated that HOXA-AS2 can regulate the proliferation of AML. As we all know, AML is mainly caused by blocked differentiation. We used a series of experiments to prove the effect of HOXA-AS2 on differentiation. Many studies have shown that CD11b and CD14 are classic differentiation markers of leukemia^29,30^. Flow cytometry showed that knockdown of HOXA-AS2 could induce the differentiation of AML cells (Fig. 3a). On the contrary, overexpression of HOXA-AS2 decreased the proportion of differentiated cells in AML cells compared with control cells (Fig. 3b). Subsequently, western blotting showed that overexpression of HOXA-AS2 decreased the expression of CD11b and CD14 in AML cells (Fig. 3c), whereas silencing HOXA-AS2 resulted in increased expression of CD11b and CD14 (Fig. 3d). Nitroblue tetrazolium (NBT) reduction showed that HOXA-AS2 knockdown significantly induced AML cell differentiation compared with the NC group (Fig. 3e). In addition, shRNA knockdown of HOXA-AS2 could promote the expression of CD11b and CD14 (Fig. 3f). In conclusion, these data suggested that HOXA-AS2 can not only regulate the proliferation of AML but also regulate the differentiation of AML.

### HOXA-AS2 regulated LATS2 transcription in AML Cells

**Fig. 4:**
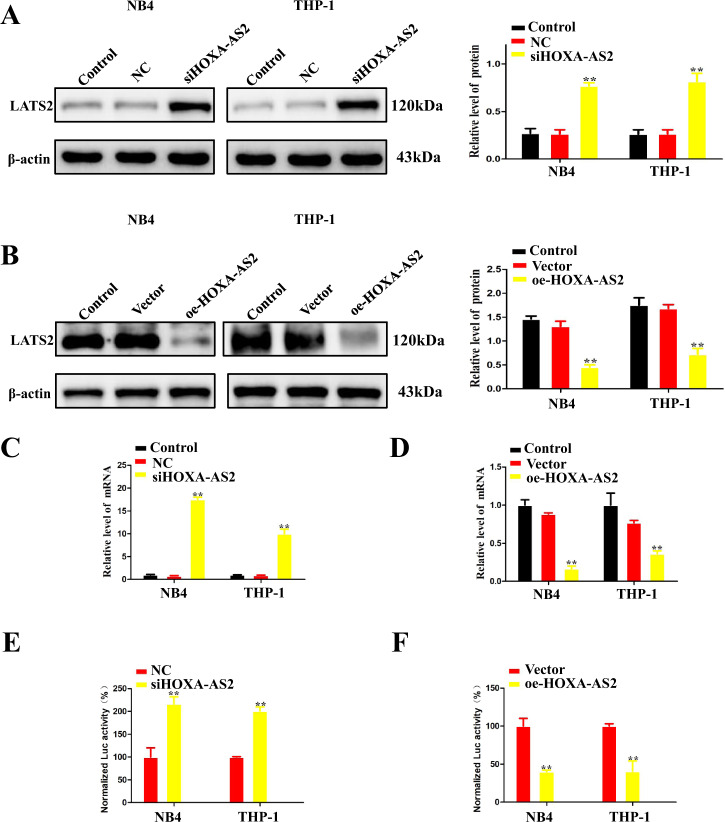
Upregulation of HOXA-AS2 inhibited LATS2 transcription in AML cells. **a**, **b** The LATS2 protein level was determined by western blot in NB4 and THP-1 cells transfected with HOXA-AS2 plasmid or transfected with si-HOXA-AS2#1, si-HOXA-AS2#2. **c**, **d** The LATS2 mRNA level was determined by qRT-PCR in NB4 and THP-1 cells transfected with HOXA-AS2 plasmid or transfected with si-HOXA-AS2#1, si-HOXA-AS2#2. **e**, **f** LATS2 transcriptional activities of NB4 and THP-1 cells transfected with HOXA-AS2 plasmid or transfected with si-HOXA-AS2#1, si-HOXA-AS2#2. *P < 0.05, **P < 0.01 versus control groups.

In order to study the role of LATS2 in AML, we next explored the relationship between HOXA-AS2 and LATS2. As shown in Fig. 4a, silencing HOXA-AS2 significantly increased LATS2 protein levels compared with negative controls. On the contrary, overexpression of HOXA-AS2 significantly decreased the expression of LATS2 (Fig. 4b). The results of gene-level showed that inhibition of HOXA-AS2 could promote the expression of LATS2 mRNA (Fig. 4c), and overexpression of HOXA-AS2 could inhibit the expression of LATS2 mRNA in AML cells (Fig. 4d). To further elucidate the specific mechanism of HOXA-AS2-regulating LATS2 expression, we used a human LATS2 luciferase reporter gene plasmid for the luciferase reporter gene analysis. Knocking down of HOXA-AS2 can increase LATS2 promoter activity in AML cells (Fig. 4e), whereas overexpression of HOXA-AS2 can also decrease LATS2 promoter activity in AML cells (Fig. 4f). These results suggested that the transcription of LATS2 in AML cells can be regulated by HOXA-AS2.

### HOXA-AS2 regulated transcriptional expression of LATS2 through recruitment of EZH2 in AML cells

**Fig. 5:**
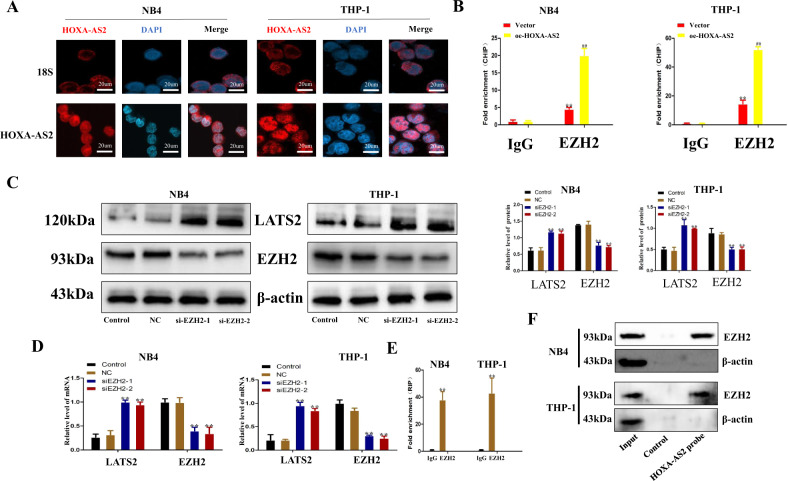
HOXA-AS2 downregulated transcriptional expression of LATS2 through recruitment of EZH2 in AML cells. **a** HOXA-AS2 distribution in NB4 and THP-1 cells was detected by FISH. **b** HOXA-AS2 plasmid was transfected into NB4 and THP-1 cells, and chrome immunoprecipitations were performed by using specific anti-EZH2 antibodies. **c** NB4 and THP-1 cells transfected with EZH2 siRNAs or the control siRNA for 72 h were collected, and EZH2 and LATS2 protein levels were detected by western blot assay. **d** EZH2 siRNAs (EZH2-1, 2) or the control siRNA were transfected into NB4 and THP-1 cells for 48 h, EZH2, and LATS2 mRNA levels were then assessed by qRT-PCR. **e** RNA immunoprecipitations were performed in NB4 and THP-1 cells, and the relative quantities of HOXA-AS2 were detected by qRT-PCR assay, normalized to the input groups. IgG and EZH2 represented the group’s coprecipitation with IgG protein and anti- EZH2 antibody respectively. **f** Total proteins were extracted from NB4 and THP-1 cells, and then HOXA-AS2 pull-down assay was performed. The EZH2 protein levels were evaluated by western blot. HOXA-AS2 probe represented the biotin-labeled HOXA-AS2 probe group and control stood for the oligo probe group. *P < 0.05, **P < 0.01 versus control groups.

The expression of HOXA-AS2 was further confirmed by FISH. The nucleus was labeled with DAPI, and HOXA-AS2 was labeled with Cy3 and 18S rRNA (cytoplasmic positive). The results showed that HOXA-AS2 was mainly located in the nuclei of AML cells (Fig. 5a). The next CHIP analysis results showed that overexpression of HOXA-AS2 can improve its binding ability to EZH2 (Fig. 5b). Many literatures have shown that EZH2 may be a key regulator of AML, therefore we silenced EZH2 expression using EZH2 siRNA. RT-qPCR and western blot results showed that the expression of EZH2 and LATS2 was negatively correlated, and silencing EZH2 could promote the expression of LATS2 (Fig. 5c, d). Subsequently, RNA immunoprecipitation (RIP) analysis showed that EZH2 protein could bind to HOXA-AS2 (Fig. 5e). The direct interaction between EZH2 and HOXA-AS2 was further demonstrated by applying the total protein (Fig. 5f) to the HOXA-AS2 pull-down assay. Taken together, we concluded that HOXA-AS2 regulated transcriptional expression of LATS2 through recruitment of EZH2 in AML cells.

### LATS2 silencing potentially involves the oncogenic function of HOXA-AS2

**Fig. 6:**
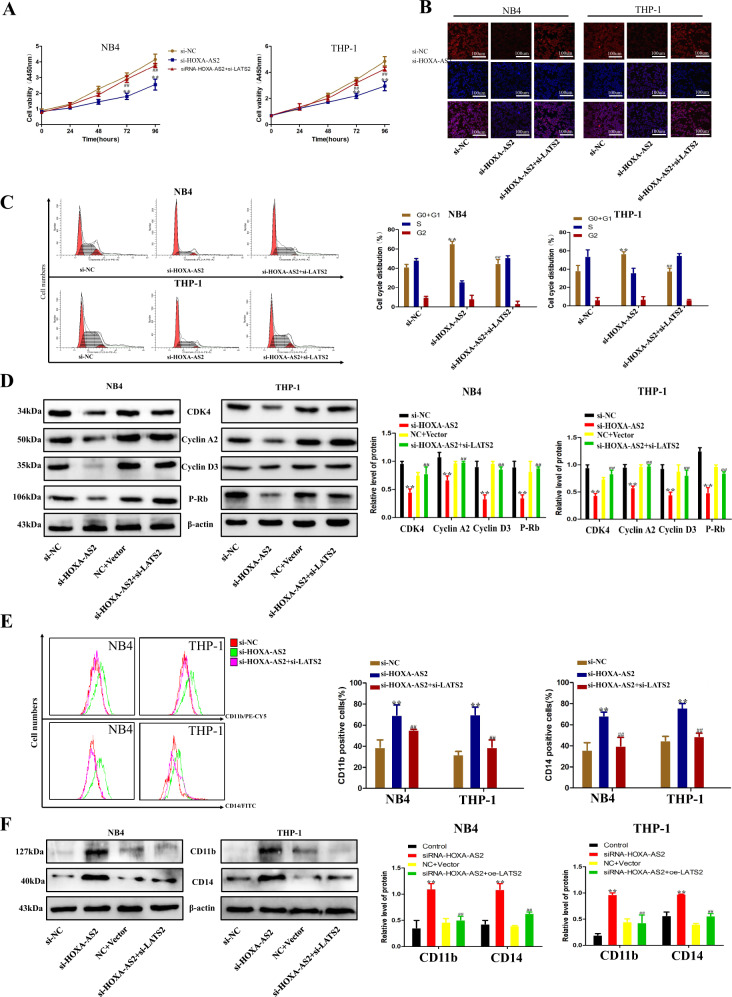
Silencing LATS2 potentially involves the oncogenic function of HOXA-AS2. **a** CCK8 assay was performed to determine the cell viability for si-HOXA-AS2 and si-LATS2 co-transfected NB4 and THP-1 cells. **b** The level of ki67 protein in NB4 and THP-1 cells co-transfection with si-HOXA-AS2 and si-LATS2 was detected by immunofluorescence. **c** The cell cycle of transfected NB4 and THP-1 cells. **d** Western blot analysis of cyclin D3, cyclin A2, P-rb, and CDK4 after si-HOXA-AS2 and si-LATS2 co-transfection in NB4 and THP-1 cells. β-actin protein was used as an internal control. **e** The levels of CD11b and CD14 protein in NB4 and THP-1 cells co-transfection with si-HOXA-AS2 and si-LATS2 were detected by flow-cytometry analysis. **f** Western blot analysis of CD11b and CD14 after si-HOXA-AS2 and si-LATS2 co-transfection in NB4 and THP-1 cells. β-actin protein was used as an internal control. ^∗^*P* < 0.05, ^∗∗^*P* < 0.01, compared with the NC siRNA group. ^#^*P* < 0.05, ^##^*P* < 0.01, compared with the si-HOXA-AS2 group.

Furthermore, to validate whether HOXA-AS2 regulates AML cell proliferation by silencing LATS2 expression, rescue assays were performed. AML cells were co-transfected with si-HOXA-AS2 and si- LATS2, and the CCK8 assay results indicated that co-transfection partially rescued si-HOXA-AS2-damaged proliferation ability (Fig. 6a). In addition, the ki67 was determined by immunofluorescence staining analysis also confirmed this result (Fig. 6b). Furthermore, flow-cytometry analysis showed that the decreased LATS2 expression reversed the G0/G1 arrest induced by HOXA-AS2 silencing (Fig. 6c). Moreover, the western blot results indicated that co-transfection increased the downregulated expression of cyclin D3, cyclin A2, P-Rb, and CDK4 triggered by the knockdown of HOXA-AS2 (Fig. 6d). Flow-cytometry analysis showed that the decreased LATS2 expression reversed the higher level of differentiation resulted from HOXA-AS2 silencing (Fig. 6e). Moreover, the western blot results indicated that co-transfection decreased the upregulated expression of CD11b and CD14 induced by the knockdown of HOXA-AS2 (Fig. 6f). These results indicated that the effect of HOXA-AS2 on AML partially involves targeting LATS2.

### Knockdown of HOXA-AS2 inhibits AML tumorigenesis in vivo

**Fig. 7:**
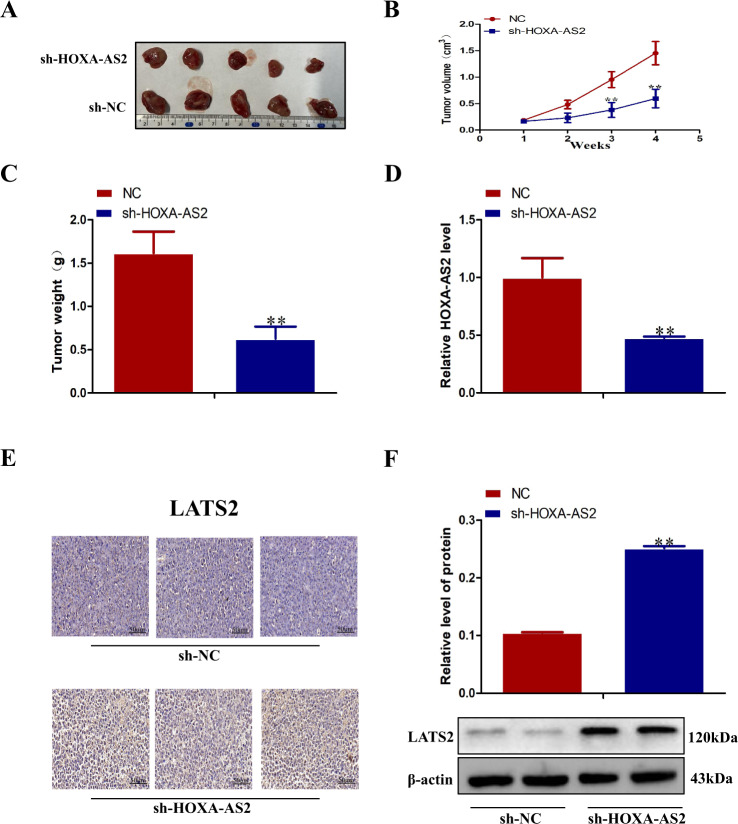
Knockdown of HOXA-AS2 inhibits AML tumorigenesis in vivo. **a** The total number of tumors after removal from the mice. **b** The tumor volumes were calculated every week after inoculation. **c** The tumor weights after the tumors were harvested. **d** qRT-PCR analyses indicated that the HOXA-AS2 expression was significantly increased in vivo. **e** Then, the protein was extracted from the tumor tissues, and expressions of LATS2 protein was detected by western blot. **f** Immunohistochemistry analysis of LATS2 was obtained from tumors. β-actin served as a loading control. ^∗^*P* < 0.05, ^∗∗^*P* < 0.01, compared with the NC shRNA group.

We have studied the in vitro study of HOXA-AS2 in AML cells, and the biological function of HOXA-AS2 in vivo. To further study the carcinogenic effect of HOXA-AS2 in AML, the control cells and shRNA HOXA-AS2 NB4 cells were injected into nude mice. Tumor volume was measured every week after injection, and the tumor tissue was removed 4 weeks later. The results showed that compared with the control group, silencing HOXA-AS2 significantly inhibited the tumor growth (Fig. 7a, b). The tumor weight of the HOXA-AS2 knockout group was significantly lower than that of the control group (Fig. 7c). Moreover, qRT-PCR analysis showed that the expression level of HOXA-AS2 in tumors was lower than that after shRNA transfection (Fig. 7d). In addition, immunohistochemistry and western blot showed that HOXA-AS2 upregulated LATS2 protein (Fig. 7e, f). In conclusion, these data suggested that HOXA-AS2 may affect the proliferation and differentiation of AML tumors in vivo, as well as in vitro experiments.

## Discussion

With the continuous improvement and progress of experimental technology, more and more new lncRNAs have been discovered by RNA sequencing technology, which plays a main role in the occurrence and development of diseases and malignant progress^31–33^. Many studies have shown that many lncRNAs express differently in AML and can regulate their functions^27,34–36^. However, whether the function and molecular mechanism of many lncRNAs are associated with AML is still unclear.

HOXA-AS2, as a new 1048 bp lncRNA, was first reported to act as an inhibitor of apoptosis in NB4 promyelocytic leukemia cells treated with all-trans-retinoic acid^19^, and has been reported to be associated with many types of malignant tumors^37^. Studies have shown that the high expression of HOXA-AS2 may be related to various biological processes of malignant tumors, such as apoptosis, invasion, migration, proliferation, and so on^38–41^, to be associated with that HOXA-AS2 is highly expressed in AML tissues and cell lines. The predicted results from the database website (GEPIA) showed that the expression level of HOXA-AS2 was negatively related to the survival of AML patients. In this study, we found that silencing HOXA-AS2 in AML cells inhibited cell proliferation and induced differentiation. Overexpression of HOXA-AS2 had the opposite biological function. In vivo experiments also showed that knockout of HOXA-AS2 can inhibit tumor growth.

As a highly conserved histone methyltransferase, EZH2 is a subunit of PRC2, which plays a regulatory role mainly by triggering H3K27me3 trimethylation^42^. It can inhibit the translation of many target genes and regulate cell cycle regulation, aging, cell proliferation, differentiation, apoptosis, and tumorigenesis^43,44^. Many studies have shown that lncRNA binds with EZH2 to exert its biological functions by regulating the expression of downstream genes^45,46^. Our recent results showed that ATPR can upregulate the expression of lncRNA NR-104098 and inhibit the differentiation and proliferation of AML^27^. The in-depth exploration revealed that lncRNA NR-104098 played a role by enhancing the binding of E2F1 to EZH2 promoter^27^. Our results indicated that HOXA-AS2 can directly bind to EZH2 and inhibit the proliferation and induce differentiation of AML by regulating the expression of LATS2.

As a confirmed tumor suppressor^47^, LATS2 plays an anti-tumor role through different signaling pathways^48–50^. Our ChIP results confirmed that EZH2 can directly bind to LATS2 promoter region in AML to regulate its expression. In addition, further rescue experiments showed that HOXA-AS2 played its biological function through LATS2. The silence of LATS2 can reverse this behavior.

## Conclusions

In conclusion, the upregulation of HOXA-AS2 may be associated with the negative prognosis of AML patients. This study first reported the high expression of HOXA-AS2 in AML tissues and cells. Silencing HOXA-AS2 can inhibit the tumorigenesis of nude mice, inhibit its proliferation, and induce its differentiation in AML cells. Moreover, HOXA-AS2 inhibited the expression of LATS2 by binding with EZH2. Our findings suggested that HOXA-AS2 may be a new target for AML therapy, and provide new hope for the diagnosis and treatment of AML targeting lncRNA.

## Data Availability

The datasets used in this study are available from the corresponding author on reasonable request.
